# Matricellular proteins in cutaneous wound healing

**DOI:** 10.3389/fcell.2022.1073320

**Published:** 2022-11-24

**Authors:** Claudia Griselda Cárdenas-León, Kristina Mäemets-Allas, Mariliis Klaas, Heli Lagus, Esko Kankuri, Viljar Jaks

**Affiliations:** ^1^ Department of Cell Biology, Institute of Molecular and Cell Biology, University of Tartu, Tartu, Estonia; ^2^ Department of Plastic Surgery and Wound Healing Centre, Helsinki University Hospital, University of Helsinki, Helsinki, Finland; ^3^ Department of Pharmacology, Faculty of Medicine, University of Helsinki, Helsinki, Finland; ^4^ Dermatology Clinic, Tartu University Clinics, Tartu, Estonia

**Keywords:** extracellular matrix, Skin, regeneration, non-structural matrix proteins, re-epithelialization

## Abstract

Cutaneous wound healing is a complex process that encompasses alterations in all aspects of the skin including the extracellular matrix (ECM). ECM consist of large structural proteins such as collagens and elastin as well as smaller proteins with mainly regulative properties called matricellular proteins. Matricellular proteins bind to structural proteins and their functions include but are not limited to interaction with cell surface receptors, cytokines, or protease and evoking a cellular response. The signaling initiated by matricellular proteins modulates differentiation and proliferation of cells having an impact on the tissue regeneration. In this review we give an overview of the matricellular proteins that have been found to be involved in cutaneous wound healing and summarize the information known to date about their functions in this process.

## 1 Introduction

**FIGURE 1:**
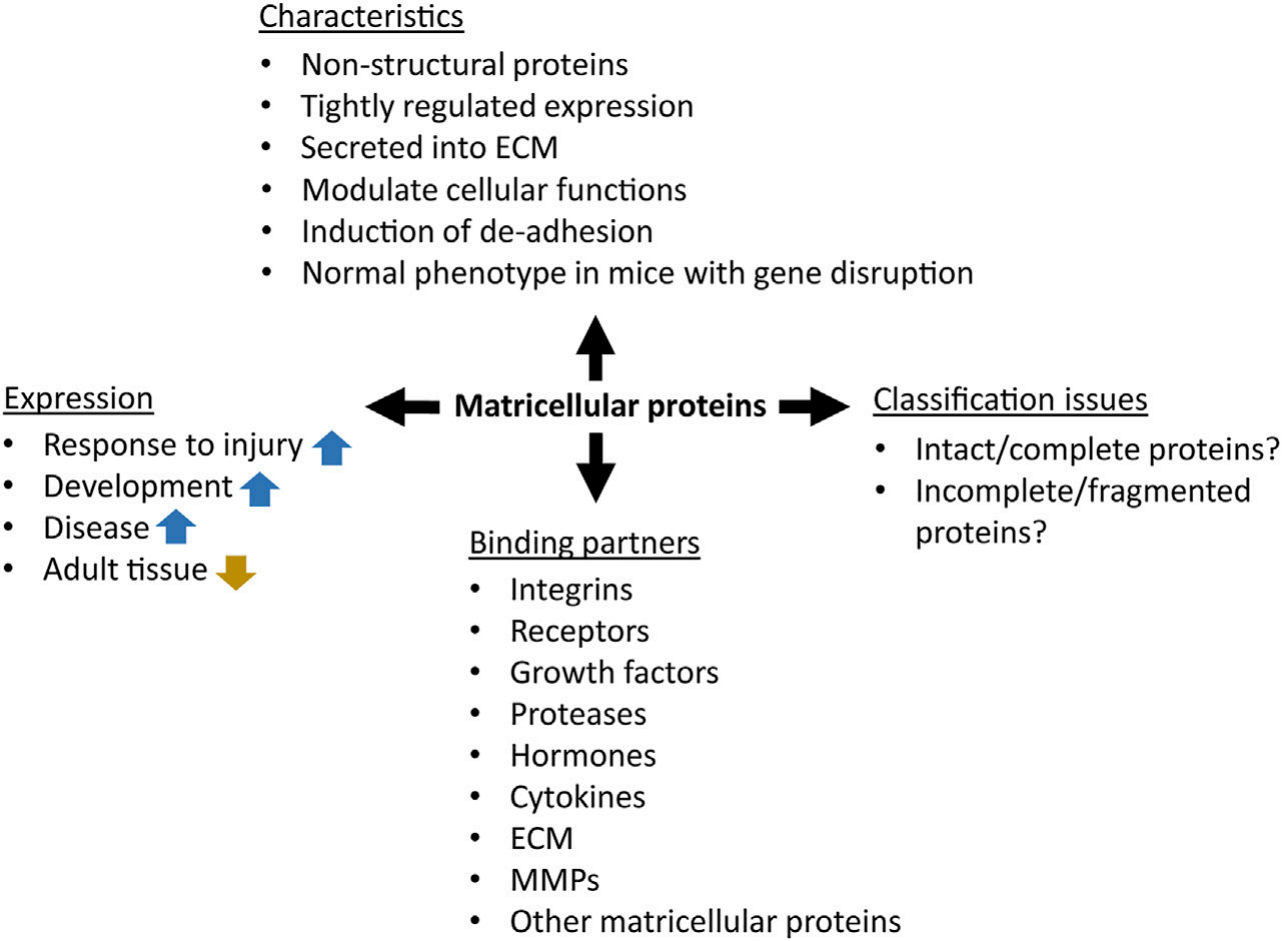
The concept of matricellular proteins. The blue and gold arrows represent upregulation and downregulation, respectively.

The term “matricellular protein” was defined by Bornstein in 1995 as follows: “a group of modular, extracellular proteins whose functions are achieved by binding to matrix proteins as well as to cell surface receptors, or to other molecules such as cytokines and proteases that interact, in turn, with the cell surface”. Matricellular proteins are secreted into the extracellular matrix, and although they can bound to structural ECM components such as collagen fibrils or basement membrane, it is assumed that they do not contribute to their mechanical functions (Figure 1) (Bornstein, 1995; Bornstein, 2009; Feng et al., 2019). In contrast to the continuous presence of structural proteins in the ECM, the expression of matricellular proteins is tightly regulated to precisely tune their functions during tissue maintenance and repair (Nikoloudaki et al., 2020). It is noteworthy that matricellular proteins are expressed at high levels during development, but their expression drops to very low levels in the adult homeostatic tissues. However, the expression of a range of matricellular proteins is activated during regeneration of tissue injury, inflammation, cancer and other pathologies (Midwood et al., 2004). This is the case for many organs like liver, kidney, skin, eye, and oral mucosa (Arriazu et al., 2014; Chatterjee et al., 2014; Kim K. H. et al., 2018; González-González and Alonso, 2018; Nyström and Bruckner-Tuderman, 2019; Theocharis et al., 2019; Nikoloudaki, 2021; Rayego-Mateos et al., 2021; Sonnenberg-Riethmacher et al., 2021).

The human cutaneous wound healing process goes through four stages: hemostasis, inflammation, proliferation and remodeling (Amiri et al., 2022). First, blood clotting occurs, during the second phase immune cells are recruited to degrade necrotic tissue, phagocyte pathogens, and secrete growth factors, chemokines, and cytokines (Reinke and Sorg, 2012). During the proliferative phase local fibroblasts migrate, proliferate, and form granulation tissue, while keratinocytes and epidermal stem cells drive the re-epithelialization by proliferation and migration towards the de-epithelialized areas of the wound (Reinke and Sorg, 2012; Li et al., 2022). The new dermal vascular network is formed, and a high amount of collagen and extracellular matrix is produced to provide a scaffold for cell adhesion, growth, movement, and differentiation of the cells within the wound. Finally, during the remodeling phase the components of the initial extracellular matrix (e.g., collagen III) are replaced with those of the scar tissue (e.g., collagen I), the wound is contracted, and wound metabolic activity stops (Reinke and Sorg, 2012). The probability of chronic wounds or fibrotic conditions in the skin increases when the re-epithelialization is interrupted or the fine balance between extracellular matrix deposition and degradation is disrupted (Rousselle et al., 2019; Amiri et al., 2022). Diverse and highly plastic macrophage populations are critically involved throughout the wound healing process by providing signal molecules for promoting and resolving inflammation, supporting cell proliferation and finally orchestrating tissue restoration (Kim and Nair, 2019; DiPietro et al., 2021). Accumulating research unequivocally demonstrates that aberrant macrophage functionality is a common feature of poorly healing and non-healing wounds.

**FIGURE 2:**
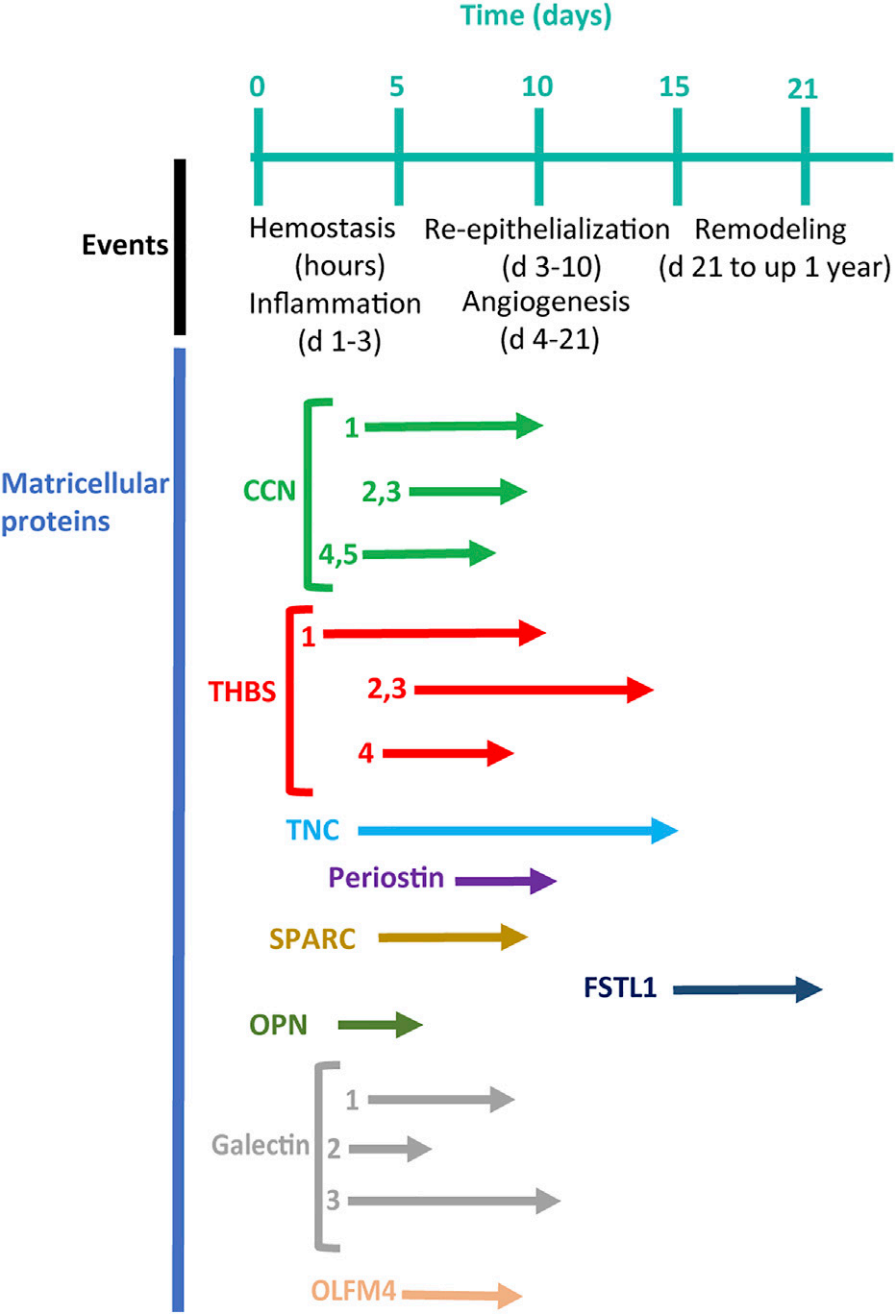
A timeline of cutaneous wound healing (Reinke and Sorg, 2012) in connection with the timing of the expression of matricellular proteins (arrows) (modified, from Midwood et al., 2004).

While a vast amount of literature is devoted to the extracellular matrix proteins (in August 2022 PubMed search with the term “extracellular matrix proteins” yielded 232,084 hits) a much smaller number of PubMed records—1838 - contained the term “matricellular”. During the 27 years, since the term “matricellular” was conceived, the categorization of matricellular proteins, particularly whether to include only intact proteins or also proteins fragments, is still under discussion (Bornstein, 2009). Although the matricellular proteins are classified by the presence of specific characteristics, and they are not always categorized by the similarity of their amino-acid sequence or common evolutionary origin. In this work, we follow the classical way of protein classification and follow the course of protein families while discussing the role of specific matricellular proteins in cutaneous wound healing. Nevertheless, it is important to mention that not all members of a specific protein family meet the criteria of matricellular protein as is the case for ENPP and fasciclin families. While preparing this review we found that 27 proteins, which match the definition of matricellular proteins have been shown to have a role in cutaneous wound healing and these belong to 11 protein families (Table 1; Figure 2). However, other emerging matricellular members, such as PAI-1 or SERPINE1 (Jayakumar et al., 2017), TINAGL1 (Sato et al., 2022), LTBP-binding proteins (Ramaswamy et al., 2019), among others (Jayakumar et al., 2017; Ramaswamy et al., 2019), were not addressed in this review, and deserve a separate discussion.

**TABLE 1:** Matricellular proteins classified by the protein family. Role in wound healing is shown, if known. For proteins with an unknown role in skin wound healing, at least one promoting/inducing function is listed, unless otherwise noted.

Protein family	Proteins	Role in skin wound healing	References	Roles of proteins with unknown role in skin wound healing
CCN	CCN1	Keratinocyte proliferation and migration, ECM remodeling, angiogenesis, inflammation, cell-matrix interactions	Chen et al. (2001); Lin et al. (2005); Minhas et al. (2011); Du et al. (2018)	
	CCN2	Angiogenesis, pro-collagen production	Igarashi et al. (1993); Lin et al. (2005); Minhas et al. (2011)	
	CCN3	Keratinocyte proliferation, angiogenesis	Lin et al. (2005); Minhas et al. (2011); Rittié et al. (2011)	
	CCN4	Fibroblast proliferation and migration	Ono et al. (2018)	
	CCN5	Keratinocyte differentiation	Rittié et al. (2011)	
	CCN6	Unknown	-	Cancer, cell motility (Barreto et al., 2016)
THBS	THBS1	Macrophage migration, endothelial cell proliferation inhibition, keratinocyte migration	DiPietro et al. (1996); Streit et al. (2000); Siriwach et al. (2022)	
	THBS2	Modulates fibroblast-matrix interaction, inhibits endothelial cell proliferation	Kyriakides et al. (1999b); Agah et al. (2005)	
	THBS3	Is active in the process, but its role is unknown	Bergmeier et al. (2018)	Chen et al. (2022b)
	THBS4	Fibroblast migration and keratinocyte proliferation	Klaas et al. (2021)	
	THBS5	Unknown	-	Cancer, cell motility (Zhang et al., 2021)
Tenascin	TN-C	Survival of implanted cells in wound beds	Forsberg et al. (1996); Rodrigues et al. (2013); Bari et al. (2020)	
	TN-X	Enhance the biomechanical properties of matrix	Egging et al. (2007)	
	TN-R	Unknown	-	Central nervous system lesion, limits synaptic plasticity (Roll and Faissner, 2019)
	TN-W	Unknown	-	Cancer, angiogenesis (Martina et al., 2010)
Fasciclin	Periostin	Fibroblast and keratinocyte proliferation and differentiation	Elliott et al. (2012); Ontsuka et al. (2012); Nikoloudaki et al. (2020); Yin et al. (2020)	
	TGFBI	Proliferation of epidermal stem cells	Li et al. (2022)	
SPARC	SPARC	Fibroblast migration, collagen fiber assembly	Basu et al. (2001); Bradshaw et al. (2002); Chun et al. (2022)	
	Hevin	Inhibits fibroblast migration by modulating adhesive interactions, and modulate ECM assembly and may suppress inflammation	Barker et al. (2005); Sullivan et al. (2008)	
	SMOC1	Angiogenesis	Luo et al. (2020)	
	SMOC2	Unknown	-	Fibrosis, induce proliferation and migration of myofibroblast (Wang et al., 2021)
	FSTL1	Unclear, improve keratinocyte growth *ex vivo*	Wankell et al. (2001); Sundaram et al. (2013)	
	SPOCK1	Unknown	-	Cancer, mediate epithelial-mesenchymal transition (Sun et al., 2020)
	SPOCK2	Unknown	-	Cancer, induce metastasis (Ren et al., 2019)
	SPOCK3	Unknown	-	Genetic risk for attention-deficit/hyperactivity disorder (Weber et al., 2014)
SIBLING	OPN	Promote fibrosis	Liaw et al. (1998); Mori et al. (2008); Buback et al. (2009); O'Loughlin et al. (2013); Chimento et al. (2017); Wang et al. (2017)	
	BSP	Unknown	-	Cancer, promote proliferation (Kruger et al., 2014)
	DMP1	Unknown	-	Chronic kidney disease, prevent bone disorder (Martin, 2019)
	DSPP	Unknown	-	Genetic risk for dental malformations (Lee et al., 2013)
	MEPE	Unknown	-	Growth plate chondrocyte matrix mineralization (Staines et al., 2012)
Galectin	Galectin-1	Activation, migration, and proliferation of myofibroblasts	Lin et al. (2015); Kirkpatrick et al. (2021)	
	Galectin-2	Is active in the process, but its role is unknown	Gál et al. (2011)	Suppresses contact allergies, proapoptotic effector for T cells (Sturm et al., 2004)
	Galectin-3	Keratinocyte migration	Liu et al. (2012); Walker et al. (2016); Gál et al. (2021)	
	Galectin-4	Unknown	-	Cancer, resistance to nutrient starvation (Huflejt and Leffler, 2004)
	Galectin-7	Keratinocyte proliferation, apoptosis and migration	Gendronneau et al. (2008)	
	Galectin-8	Unknown	-	Angiogenesis, promote endothelial cell migration (Troncoso et al., 2014)
	Galectin-9	Unknown	-	Autoimmune disorders (Moar and Tandon, 2021)
	Galectin-10	Unknown	-	Immune system diseases (Su, 2018)
	Galectin-12	Unknown	-	Energy homeostasis (Yang et al., 2012)
	Galectin-13	Unknown	-	Protect pregnancy (Sammar et al., 2019)
	Galectin-14	Unknown	-	Immune tolerance during pregnancy (Si et al., 2021)
R-spondin	RSPO1	Proliferation and differentiation of keratinocytes	Kuppan et al. (2011); Sundaramurthi et al. (2012)	
	RSPO2	Unknown	-	Cancer, promote proliferation (Sun et al., 2021b)
	RSPO3	Unknown	-	Cancer, proliferation (Hilkens et al., 2017)
	RSPO4	Unknown	-	Genetic risk for autosomal-recessive anonychia (Brüchle et al., 2008)
Fibulin	Fibulin 1	Unknown	-	Beneficial marker for preeclampsia (Ustunyurt et al., 2021)
	Fibulin 2	Unknown	-	Cancer, unclear (Zhang et al., 2020a)
	Fibulin 3	Unknown	-	Cancer, promotes epithelial-mesenchymal transition (Zhou et al., 2020)
	Fibulin 4	Unknown	-	Cancer, promotes proliferation (Li and Wang, 2016)
	Fibulin 5	Collagen expression	Lee et al. (2004)	
ENPP	ENPP2	Keratinocyte migration	Mazereeuw-Hautier et al. (2005); Ryu and Han, (2015)	
OLFM	OLFM1	Unknown	-	Axonal growth (Nakaya et al., 2012)
	OLFM2	Unknown	-	Genetic risk of ocular anomalies (Holt et al., 2017)
	OLFM3	Unknown	-	Epilepsy (Tang et al., 2020)
	OLFM4	Keratinocyte proliferation and migration, fibroblast migration	Klaas et al. (2022)	
	ADGRL1	Unknown	-	Behavioral abnormalities (Vitobello et al., 2022)
	ADGRL2	Unknown	-	Genetic risk for extreme microcephaly (Vezain et al., 2018)
	ADGRL3	Unknown	-	Genetic risk for attention-deficit/hyperactivity disorder (Kappel et al., 2019)
	Myocilin	Unknown	-	Genetic risk for glaucoma (Fingert et al., 2002)
	Gliomedin	Unknown	-	Genetic risk for peripheral neuropathies (Maluenda et al., 2016)
	OLFML1	Unknown	-	Genetic risk for abnormal bone development (Murakami et al., 2018)
	OLFML2A	Unknown	-	Cancer, required for AP-1 overexpression (Zhao et al., 2021)
	OLFML2B	Unknown	-	Cancer, biomarker (Lin et al., 2021)
	OLFML3	Migration endothelial cells	Dunn et al. (2016)	Cancer, unclear (Stalin et al., 2021)

Although the roles of matricellular proteins in cutaneous wound healing have been discussed earlier (Walker et al., 2016) we felt that bringing the subject into the scientific spotlight might inspire further research in this area that may result in better treatment options for both acute and chronic wounds. Since the main source of knowledge on cutaneous wound healing comes from murine models, we refer to results from murine models in this review, unless otherwise stated.

## 2 Functions of matricellular proteins in cutaneous wound healing

### 2.1 Cellular communication network factor family

The CCN family is a group of secreted proteins that specifically associate with other extracellular matrix proteins and consists of 6 members: CCN1 (cysteine-rich 61, CYR61), CCN2 (connective tissue growth factor, CTGF), CCN3 (overexpressed in nephroblastoma, NOV), CCN4 (Wnt-1 induced secreted protein-1, WISP1), CCN5 (Wnt-1 induced secreted protein-2, WISP2) and CCN6 (Wnt-1 induced secreted protein-3, WISP3) (Brigstock, 2003). Each CCN protein is comprised of four distinct functional domains, which have significant homology and functional similarity to other proteins and growth factors (Rachfal and Brigstock, 2005). Although the primary structures of CCN proteins are very similar, their three-dimensional structures differ significantly, resulting in marked differences in their interaction partners and distinct functions (Sun C. et al., 2021). CCN proteins play a role in angiogenesis, wound healing, tumor growth, placentation, implantation, embryogenesis, endochondral ossification, and inflammation (Schutze et al., 2005; Leask, 2008; Lemaire et al., 2010; Lin et al., 2010; Kim H. et al., 2018). For these reasons CCN proteins were initially defined as growth factors that regulate cell proliferation, adhesion, mitosis, migration, growth arrest, apoptosis, differentiation, and ECM production (Brigstock, 2003; Perbal, 2004; Leask and Abraham, 2006; Kubota and Takigawa, 2007; Quan et al., 2010; Kular et al., 2011; Quan et al., 2012; Sun C. et al., 2021). Functionally, these proteins serve as adaptor molecules that connect cell surface and extracellular matrix. They execute their functions by modulating the activity of several growth factors, thus can act indirectly (e.g. binding TGF-β), but can also act directly (e.g. binding to integrins) (Leask and Abraham, 2006; Holbourn et al., 2008; Lau, 2016).

Transcriptomics analysis of healthy skin revealed that CCN5 was the most abundantly expressed member of CCN family, followed by CCN2, CCN3, and CCN1. CCN4 and CCN6 proteins are expressed at relatively low levels in human skin (Grzeszkiewicz et al., 2001; Quan et al., 2009; Rittié et al., 2011). Altered CCN gene expression is associated with numerous pathological conditions, including fibrotic disorders and tumorigenesis (Bleau et al., 2005; Leask and Abraham, 2006; Lemaire et al., 2010; Riser et al., 2015; Perbal, 2018). However, as the functions of CCN proteins are governed by the bioavailability of their multiple interaction partners such combinatorial effects can be difficult to decipher (Perbal, 2001; Holbourn et al., 2008). Differences in temporal and locational patterns of expression and interactions between the CCN family members can further add to these challenges. To this background, it is therefore perhaps not even surprising that conflicting results have sometimes been reported.

Rittié et al., found that the expression of CCN transcripts is generally higher in the dermis than in the epidermis. In dermis, CCN2, CCN3 and CCN5 proteins were mainly expressed in fibroblasts, blood vessels, eccrine sweat glands, and hair follicles, whereas in upper dermis CCN5 protein was mainly associated with reticular fibers. As both CCN3 and CCN5 levels decreased during re-epithelialization, these proteins are thought to regulate the proliferation/differentiation status of epidermal keratinocytes (Rittié et al., 2011). Minhas et al. investigated the expression pattern of CCN1, CCN2 and CCN3 in human acute and chronic wounds. They found that the amount of CCN1 protein was comparable in normal skin and acute wounds, while in chronic wounds its level was significantly increased. In normal skin the level of CCN2 and CCN3 proteins was similar and lower than that of CCN1 (Minhas et al., 2011).

CCN1 fulfils critical task in cutaneous wound healing, e.g., stimulates the removal of apoptotic neutrophils by macrophage and facilitates the progression of wound healing process from the inflammatory to the proliferative phase. Nevertheless, since CCN1 induce different functions in distinct cell types at various stages of cutaneous wound healing, its inhibition or overexpression may have different consequences depending on the nature of the damage, and timing of perturbation (Kim K. H. et al., 2018). Du et al. (2018) showed that CCN1 was upregulated during the early stages of cutaneous wound healing, and promoted keratinocyte migration and proliferation. Intradermal treatment with CCN1 accelerated wound closure and re-epithelialization in a full-thickness and superficial second-degree burns mice models (Du et al., 2018). CNN2 is required for ECM-evoked signaling in fibroblasts during active tissue remodeling. Its downregulation impedes wound healing and the lack of signals evoked from ECM in fibroblasts leads to disruption of normal regeneration, downregulation of CTGF and appearance of chronic wounds (Minhas et al., 2011). The expression of CCN3 during cutaneous wound healing correlates with transforming growth factor (TGF) -β1 levels at 5–7 days after wounding, and CCN3 has been suggested to exert its effects through interaction with yet unknown integrins by increasing DNA synthesis, supporting fibroblast adhesion, and inducing fibroblast chemotaxis (Lin et al., 2005). However, the level of CCN3 expression did not differ between acute and chronic wounds (Minhas et al., 2011). CCN4 was highly expressed at post-injury stages using a mouse model of full-thickness wounds and CCN4 was shown to contribute to wound closure by regulating dermal fibroblast migration and proliferation through the integrin α5β1 and extracellular signal-regulated kinases signaling pathway (Ono et al., 2018). Studies conducted so far have not found roles for CCN5 and CCN6 in the wound healing (Quan et al., 2009; Ji et al., 2014; Leask, 2016).

Although CCN1, CCN2 and CCN3 have been shown to promote angiogenesis in corneal implants (Babic et al., 1999; Lin et al., 2003; Chen and Lau, 2009), placenta (Mo et al., 2002), chick chorioallantoic membrane (Shimo et al., 1999) and rabbit ischemic hindlimb model (Fataccioli et al., 2002) *in vivo*, little is known about their roles in this respect in cutaneous wound healing. Subcutaneous implantation of stainless-steel mesh chambers in rats showed an increase of CCN2 at transcript level at day 9, which coincided with the growth of granulation tissue suggesting its role in neovascularization during wound healing (Igarashi et al., 1993). Further, it was found that CCN1, CCN2 and CCN3 were co-expressed in dermal fibroblasts located in the granulation tissue of full-thickness incisional mouse wounds suggest a role for these proteins in ECM remodeling, wound contraction, and angiogenesis potentially *via* regulating of vascular endothelial growth factor-A and -C expression (Chen et al., 2001).

### 2.2 Thrombospondin family

Thrombospondins (THBS) comprise an evolutionarily conserved family of extracellular, oligomeric, multidomain, calcium-binding glycoproteins that interact with other extracellular matrix components and receptors on the cell surface (Adams and Lawler, 2011). These proteins contribute actively to wound healing, angiogenesis, connective tissue organization and synaptogenesis (Adams, 2001; Adams and Lawler, 2004; Bornstein et al., 2004; Zhang and Lawler, 2007; Isenberg et al., 2009). Thrombospondin family consists of five secreted proteins that have diverse roles in modulating cellular function. By now five different paralogs of thrombospondin have been identified (thrombospondins-1–5), which form either trimers (subgroup A: THBS1 and THBS2) or pentamers (subgroup B: THBS3–5). THBSs 3 to 5 lack the procollagen domain and type I repeats of the trimeric proteins and contain four rather than three type II epidermal growth factor (EGF)-like repeats differing thereby from THBS 1 and 2 (Bornstein and Sage, 1994; Adams, 2001; Adams and Lawler, 2011).

THBS1 is a secreted glycoprotein found in the ECM and pericellular matrix (DiPietro et al., 1996; Agah et al., 2002). THBS1 expression is activated by TGF-β signaling pathway among others. THBS1 binds to collagens I and V, fibronectin, laminin, fibrinogen, and secreted protein, acid and rich in cysteine proteins, regulates extracellular matrix metalloprotease (MMP) levels (e.g. MMP3, MMP9, MMP11, and MMP13) (Bornstein, 1995; Adams, 1997; Tan and Lawler, 2009). The cluster of differentiation 36 receptor on endothelial cells serves as the primary receptor for THBS1 (Iruela-Arispe et al., 1999). THBS1 can bind also other cell surface receptors such as cluster of differentiation 47 and the integrin family of ECM receptors (Adams, 1997; Isenberg et al., 2006). Thrombospondin-1 has been described as the first naturally occurring inhibitor of angiogenesis (Good et al., 1990; Iruela-Arispe et al., 1991; Lawler and Detmar, 2004). In human skin, THBS1 is secreted by basal epidermal keratinocytes and is deposited in the vicinity of basement membrane (Wight et al., 1985), contributing to the normal anti-angiogenic barrier that separates the avascular epidermis from the vascularized dermis (Detmar, 2000). Transient increase in THBS1 levels was correlated with increased cell migration during skin wound healing (DiPietro et al., 1996). However, chronic overexpression of THBS1 in the skin of transgenic mice inhibited cutaneous tissue repair, granulation tissue formation and wound angiogenesis (Streit et al., 2000). It has been shown that a subpopulation of migrating THBS1(+) keratinocytes that originated from basal keratinocytes in the wound facilitated epidermal wound healing as these migrated suprabasally toward the wound front and finally differentiated into neo-epidermis (Siriwach et al., 2022). It should be noted that controversial data on THBS1 functions have been reported over the years. A most plausible explanation is that THBS1 acts in a context-dependent manner even for the same cell type (Ichii et al., 2002; Calzada et al., 2004; Roberts, 2011).

THBS2 is primarily produced by fibroblasts and smooth muscle cells, and is implicated in the remodeling phase of tissue repair (Krady et al., 2008). THBS2 is synthesized and secreted as homotrimer. It contains N-terminal laminin G-like domain, a von Willebrand factor pro-collagen-like domain, three type 1 properdin-like repeats, three EGF-like type II repeats, seven EGF-like type III repeats, and a carboxy terminal lectin type domain (Calabro et al., 2014). These domains are involved in regulation of multiple cellular functions *via* forming multiple interactions with cell surface proteins and MMPs that have been described in detail previously (Lawler and Lawler, 2012; Murphy-Ullrich and Iozzo, 2012). Like THBS1, THBS2 is classified as an angiogenesis inhibitor by inhibiting basic fibroblast growth factor-induced vascular invasion (Simantov et al., 2005). Furthermore, increased levels of THBS2 have been observed in the blood of patients with scleroderma, suggesting the involvement of THBS2 with inflammation, vascular damage, and fibrosis of the skin and internal organs (Kajihara et al., 2012). In an excisional wound model in THBS2-null mice, cutaneous wounds presented irregular collagen organization, increased cellularity, and highly vascularized granulation tissue (Kyriakides et al., 1999a). The collagen fibrils were larger in diameter, had abnormal contours, and were disorganized, causing the lower tensile strength of the skin (Kyriakides et al., 1998). On the other hand, the THBS2-null mice had faster wound healing and minimal scarring, probably due to the better vascularization of the wound tissue (Kyriakides et al., 1999b). Whereas the inhibition of THBS2 expression results in an increase in MMP-2 activity, in the absence of THBS2, accumulates MMP-2 in the extracellular space, resulting in a significant reduction in cell adhesion (Yang et al., 2000; Yang et al., 2001; Agah et al., 2005).

Although very little is known about the role of THBS3, it has been suggested that it has common functions with other family members, especially with THBS-5. THBS-3 expression has been studied in the developing mouse (Iruela-Arispe et al., 1993), chicken (Tucker et al., 1997), *Xenopus laevis* (Lawler et al., 1993), and adult human tissues, where the highest levels of this protein were found in kidney, pituitary gland, trachea, uterus, and fetal kidney (Adolph, 1999). THBS3-null mice showed abnormalities in the postnatal skeleton, indicating that THBS3 may have a role in regulating skeletal maturation in mice (Hankenson et al., 2005). Since THBS-3, like other THBSs, binds heparin, it may be able to bind to other proteoglycans as well (Qabar et al., 1994), but direct binding of THBS-3 to ECM molecules has not been yet demonstrated. Analysis of total skin wound extracts collected at different time points after skin injury showed a slight increase in THBS3 mRNA levels in granulation tissue, which may indicate that THBS3 also plays a role in skin wound healing (Bergmeier et al., 2018).

THBS4 is a member of subgroup B of thrombospondins. THBS-4 was first identified in *Xenopus laevis* embryos as a constituent of the myotome and skeletal muscle (Urry et al., 1998). In adult human tissues, the highest expression of THBS4 is found in the heart and skeletal muscles (Lawler et al., 1993). THBS4 has been found in the basement membrane zone of the human ocular surface epithelium, a special microenvironment where stem cell maintenance, self-renewal, activation and proliferation by external action occur (Schlötzer-Schrehardt et al., 2007). THBS4 protein is produced by endothelial cells and smooth muscle cells in the vascular wall (Stenina et al., 2003). The unique spatial and temporal patterns described for THBS4 and its presence specifically in neural tissue suggest that it performs different functions than other thrombospondins. For instance, THBS4 is thought to interact directly with the ECM and promote neurite outgrowth (Arber and Caroni, 1995; Stenina-Adognravi, 2014). Similar to other THBSs, THBS4 regulates collagen expression and ECM structure formation, repair, and remodeling. In the absence of THBS4 the formation of collagen fibrils is disorganized, the number of fibrils with a larger diameter is increased, as well as space between fibrils is larger (Kyriakides et al., 1998; Frolova et al., 2010). While in lower organisms THBS4 participates in limb regeneration and in the formation of the so-called transitional matrix, in higher organisms THBS4 participates in wound healing and scar formation (Whited et al., 2011; Qian et al., 2018). Previously, we found that THBS4 expression is upregulated in the dermal part of healing skin wounds in both humans and mice through the β-catenin signaling pathway. Concordantly, the recombinant THBS4 protein promoted the healing of skin wounds in mice, stimulating the migration of primary fibroblasts and the proliferation of keratinocytes (Klaas et al., 2021).

A THBS5 monomer consists of a N-terminal domain, four EFG-like domains, eight calmodulin-like domains, and a C-terminal domain. THBS5 is secreted as pentamers that localize to ECM (Acharya et al., 2014; Kim et al., 2015). THBS5 Interacts with several ECM components suggesting that THBS5 may play an important role in establishing and maintaining ECM structure by forming a molecular bridge between different components of the matrix or between the matrix and the cell membrane (Budde et al., 2005). The expression of this protein has been found in chondrocytes, tendons, ligaments, smooth muscle cells, synovium, and osteoblasts (Riessen et al., 2001; Andrés Sastre et al., 2021).

### 2.3 Tenascin family

Tenascins are glycoproteins that belong to the class of matricellular proteins that are associated with cell motility, proliferation, and differentiation. The tenascin family is comprised of four homologous proteins: tenascin-C (Midwood et al., 2016), tenascin-R, tenascin-W and tenascin-X (Miller, 2020). The best studied and first described member of the tenascin family is tenascin-C (TN-C). TN-C is a hexameric ECM protein with highly conserved structure and multiple binding that binds directly to cell surface receptors as well as to other ECM components, soluble factors, and pathogens (Midwood et al., 2016).

The first suggestions that the tenascins could have a role in wound healing came from full-thickness mouse wound healing model that demonstrated that TN-C has a very low expression in normal healthy skin but was strongly induced in healing wounds (Mackie et al., 1988; Forsberg et al., 1996). The upregulation of TN-C during wound healing has also been reported in more recent proteomic studies where it was suggested to regulate epidermal growth factor receptor signaling and enhance the survival of mesenchymal stem cells upon *in vivo* implantation (Rodrigues et al., 2013; Bari et al., 2020). Experiments with cultured human keratinocytes showed that IL-4 acts as a major inducer of TN-C expression whereas TNFα and IFNγ moderately increased TN-C expression (Latijnhouwers et al., 1998). TN-C is also upregulated in brain tissues after stroke demonstrating its more universal role in tissue regeneration (Okada and Suzuki, 2020).

The expression pattern of TN-X is distinct from TN-C and has been shown to be tissue specific and developmentally regulated (Burch et al., 1995; Geffrotin et al., 1995). The loss of TN-X has been associated with Ehlers-Danlos Syndrome, an autosomal dominant disorder of collagen deposition that is characterized by reduced skin tensile strength and reduced skin collagen content (Burch et al., 1997). Tn-X deficient mice show a skin phenotype that is similar to Ehlers-Danlos Syndrome in humans (Mao et al., 2002). TN-X may function as an ECM organizer by binding to fibrillar collagen and stabilizing its structure acting indirectly on the cells (Lethias et al., 2006). TN-X is potentially involved in the later phase of cutaneous wound healing and it may be involved in the remodeling and maturation of matrix enhancing its biomechanical properties (Egging et al., 2007). Furthermore, *in vitro* culture of dermal fibroblasts showed that TN-X deficient cells fail to deposit collagen I into ECM (Mao et al., 2002). More recent studies using Tn-X deficient mice have shown that the loss of Tn-X prolongs corneal epithelial wound healing and increased neutrophilic inflammatory response (Sumioka et al., 2021).

No relevant information was found regarding the role of TN-R and TN-W in cutaneous wound healing. The TN-W protein is mainly expressed in periosteum of the bone tissue, developing smooth muscle, adult kidney and in stem cell niches (Scherberich et al., 2004). *In vitro* studies using a murine myoblast cell line, have shown that TN-W modulates cell adhesion by inhibiting cell spreading (Brellier et al., 2012). The role of TN-W in cell adhesion and its expression profile suggest that TN-W may have a role in tissue repair (Degen et al., 2020).

### 2.4 Fasciclin family

The fasciclin protein domain was initially identified in insects (Premachandra et al., 2013). This is an ancient structural motif present in certain extracellular proteins across all kingdoms of multicellular organism and is particularly frequently found in plants. In humans there are four proteins that contain fasciclin domain: TGF-β induced protein (TGFBI), periostin, stabilin-1 and stabilin-2 (Stab2) (Seifert, 2018). Of these TGFBI and periostin can be categorized as matricellular proteins, because they are secreted (Fico and Santamaria-Martínez, 2020; Yin et al., 2020). While Stabilin-1 and 2 function as scavenger receptors and are involved in regulating intracellular protein trafficking (Kzhyshkowska et al., 2006; Harris and Baker, 2020).

Periostin is a multifunctional glycoprotein closely associated with wound healing (Yin et al., 2020). Periostin has been shown to promote the proliferation and differentiation of skin keratinocytes and fibroblasts (Nikoloudaki et al., 2020). In agreement with this wound closure is slower in periostin-deficient mice that was accompanied with significant reduction of myofibroblasts in the wound (Elliott et al., 2012). Additionally, periostin facilitated wound healing *in vivo* by promoting the proliferation and migration of dermal fibroblasts (Ontsuka et al., 2012). Periostin can indirectly participate in the formation of the ECM, it plays an important role in the interaction between the cells and the surrounding microenvironment, *via* to TN-C, BMP-1 CCN3, proteoglycans, collagen and fibronectin, and can act directly on intracellular signaling pathways *via* binding to integrins (Wang et al., 2022).

TGFBI is a glycoprotein expressed in various tissues, including bone, cartilage, heart, liver, and skin. It participates in various physiological processes, such as differentiation, morphogenesis, cell growth, inflammation, tumor progression and metastasis (Lang et al., 2019). TGFBI is predominantly expressed in the dermis in normal skin and facilitates wound re-epithelialization by promoting the proliferation of epidermal stem cells through the classical Wnt pathway (Li et al., 2022). Earlier it has been found that TGFBI binds directly to integrin αvβ3 to exert its functions (Son et al., 2013).

### 2.5 The secreted protein, acid and rich in cysteine family

The SPARC family is one of the more widely studied matricellular protein groups. This family consists of eight members: SPARC (osteonectin, BM-40), Hevin (SPARC-like 1, MAST9), secreted modular calcium binding protein (SMOC) 1 and 2, SPOCK1 (also named testican), SPOCK2, SPOCK3 and follistatin like protein 1 (FSTL1 (also named TSC-36, flik, FRP)) (Brekken and Sage, 2000; Bradshaw, 2012). All members of the family have a follistatin-like domain, an extracellular calcium binding E-F hand motif, and are secreted into extracellular space.

SPARC proteins regulate extracellular matrix assembly and deposition, disrupt cell adhesion (act as counteradhesives), inhibit cell proliferation, regulate the activity of extracellular proteases, and modulate the activity of growth factor/cytokine signaling pathways, *via* direct interaction with the binding partners on the cell surface. These proteins are expressed in mouse embryos from day 9 of gestation. In adults the expression is limited to tissues with relatively high regenerative potential such as bone, skin and gut (Bradshaw, 2012). An abnormal expression of SPARC proteins is found in human pathologies such as chronic inflammation (Riley and Bradshaw, 2020), fibrosis (Trombetta-Esilva and Bradshaw, 2012), rheumatoid arthritis (Sangaletti et al., 2021), kidney diseases (Bao et al., 2021), diabetes (Kos and Wilding, 2010), central nervous system diseases (Chen et al., 2020), cancer and cancer metastasis (Ribeiro et al., 2014; Said, 2016; Camacho et al., 2020).

SPARC is required for granulation tissue formation during normal cutaneous wound repair in mice and regulates cell migration *in vitro* (Basu et al., 2001). However, a reduced collagen content was found in healing wounds of SPARC-knockout mice, leading to improved contractibility of wound edges and enhanced wound closure (Bradshaw et al., 2002). In accordance with this finding, the downregulation of SPARC in mouse wounds led to delayed wound contraction and reduced collagen deposition, thus potentially preventing excessive scar formation (Chun et al., 2022).

Similarly, it was found that in Hevin-null mice inflammation and fibrosis were induced in cutaneous injuries (Barker et al., 2005). Another study showed that in Hevin-null mice excisional and incisional cutaneous wound healing was enhanced by early infiltration of macrophage into the wound beds. A potential mechanism for this effect was suggested by *in vitro* experiments where Hevin inhibited the migration of primary dermal fibroblasts in Rac-1–dependent manner (Sullivan et al., 2008).

SMOC proteins are also implicated in regulation of wound healing as fastening induced SMOC1 upregulation in mouse diabetic and burn wounds was accompanied by neovascularization and accelerated healing (Luo et al., 2020). SMOC2 protein was shown be present in the basal layers of the mouse epidermis, but its role in keratinocyte homeostasis is currently unknown. Nevertheless, recombinant SMOC2 stimulated the attachment of primary epidermal cells, as well as several epidermal-derived cell lines *in vitro*. SMOC2 also stimulated migration of keratinocyte-like HaCaT cells but had no effect on their proliferation and did not alter the attachment of non-epidermal cells (Maier et al., 2008).

FSTL1 protein was expressed at readily detectable levels in the skin of mice, however, there was no change in its expression after wounding (Wankell et al., 2001). Interestingly, this protein could not be detected in chronic non-healing diabetic ulcers (Sundaram et al., 2013). This protein may still play a role in wound healing as FSTL1 promoted keratinocyte migration in an *ex vivo* human skin culture (Sundaram et al., 2013). The addition of recombinant FSTL1 to wounds decreased tissue contraction and inhibited dermal scar formation by sequestering activin B, growth differentiation factors 8, 9 and 11 and/or bone morphogenetic proteins 4, 6, 7 and 15 (Monsuur et al., 2018). The potential roles of SPOCK proteins in cutaneous wound healing have yet to be established.

### 2.6 The small integrin-binding ligand, N-linked glycoproteins family

The SIBLING family of proteins encompass small glycophosphoproteins that bind to a variety of proteins and the mineral phase of bones and teeth. The five members of SIBLING family are: osteopontin (also named OPN, SPP1), bone sialoprotein, dentine matrix protein 1, dentin sialophosphoprotein and matrix extracellular phosphoglycoprotein (also named MEPE) (Fisher et al., 2001). All SIBLING family members have a poorly conserved amino acid sequence characterized by the abundance of acidic amino acids, the presence of arginylglycylaspartic acid or RGD motif, relatively conserved motifs for post-translational modifications and at least one motif for controlled proteolysis. SIBLINGs proteins can signal directly to cells by binding to integrins, and other cell surface proteins, and can have an indirectly effect on cell behavior through regulating MMPs, and complement factor H. Thus, SIBLINGs act as adhesion modulators, as well as autocrine and paracrine signaling molecules (Bellahcène et al., 2008).

Osteopontin (OPN) is expressed in the basal keratinocyte layer, hair follicles, sebaceous glands, and sweat glands in both human and murine skin. The role of OPN in skin pathologies including chronic wounds has been well established but its role in cutaneous wound healing remains controversial (Buback et al., 2009; Chimento et al., 2017). The expression of OPN was increased in murine cutaneous wounds in wild-type mice and in OPN null mice the wound closure was notably delayed. OPN stimulated the migration of mesenchymal stem cells *in vitro* and in OPN null mice the migration of mesenchymal stem cells was negatively affected. Since the expression of cluster of differentiation 44 and its receptor E-selectin were downregulated in OPN null mice it could be speculated that these molecules mediate OPN’s effects during wound healing (Liaw et al., 1998; Wang et al., 2017). It has been shown that inflammatory signals trigger the expression of OPN in wound fibroblasts that may, at least in part mediate the emergence of fibrotic alterations (e.g., scarring) during wound healing (Mori et al., 2008). Such stimulatory properties on wound healing have pointed to OPN as a new therapeutic agent for treatment of diabetic foot ulcers (O'Loughlin et al., 2013).

SIBLING proteins are expressed mainly in bone and teeth but also in specific elements of normal ductal epithelium in the salivary gland and kidney. Furthermore, all SIBLINGs proteins except MEPE are expressed in the metabolically active epithelium of the human eccrine sweat gland duct, but unlike OPN, not in other skin compartments (Ogbureke and Fisher, 2007).

### 2.7 Galectin family

Galectins are small, soluble proteins that bind to β-galactoside and possess at least one conserved carbohydrate-recognition domain (CRD). Galectins are located in cytosol as well as in the nucleus, and are secreted to intercellular space *via* a non-classical secretion pathway that bypasses the Golgi complex (Johannes et al., 2018). Galectin family includes 15 proteins in mammals; however, four of them (galectin-5, -6, -11 and -15) are not found in humans. Based to their structure, galectins are categorized as monovalent (containing a single CRD, galectin-1, -2, -5, -7, -10, -11, -13, -14, and -15), bivalent (containing two CRD, galectin-4, -6, -8, -9, and -12), and chimeric (containing a single CRD and a unique amino terminus, galectin-3) (Elola et al., 2007). There exists a wealth of literature that addresses the role of galectins in cell adhesion, migration, inflammation, re-epithelialization, and skin physiology (Elola et al., 2007; Panjwani, 2014; Brinchmann et al., 2018; Johannes et al., 2018; Wu and Liu, 2018). Here we discuss more recent findings that describe the involvement of galectins 1, 3 and 7 in cutaneous wound healing.

Galectin-1 is upregulated in skin during the early phases of cutaneous wound healing in rats and potentially plays a role in regulating wound contraction. In galectin-1 knockout mice, cutaneous wound healing was delayed when compared to wild-type mice. Galectin-1 promoted the activation, migration, and proliferation of myofibroblasts *in vitro*. The molecular mechanisms by which galectin-1 may exert its effects include binding to neuropilin-1, and modulation of Smad3/NADPH oxidase 4 signaling (Lin et al., 2015). Simultaneously, the expression of galectin-1 was increased in the dermis during cutaneous wound healing and the amount of this protein remained high in both human and porcine hypertrophic scars. This suggests that galectin-1 may have a role in the formation of scars and deregulation of its expression may facilitate formation of hypertrophic scars by stimulating fibroblasts that result in their hyperproliferation, excessive collagen secretion, and dermal thickening (Kirkpatrick et al., 2021).

Galectin-3 promotes re-epithelialization of cutaneous wounds by stimulating cell migration but not proliferation. Keratinocytes derived from galectin-3-null mice showed impaired migration and re-epithelialization of skin wounds (Liu et al., 2012). However, the speed of wound healing was not altered in galectin-3 knockout mice despite delayed re-epithelialization, and immune cell infiltration. Additionally, the expression of genes associated with fibrotic response as well as the vascularization efficiency were not affected (Walker et al., 2016). The level of galectin-3 is slightly increased during later phases of wound healing and is coinciding with the induction of scarring (Gál et al., 2011; Gál et al., 2021). In line with this, the administration of galectin-3 to cutaneous wounds increased the tensile strength of resulting scars and improved collagen organization suggesting that galectin-3 possesses profibrotic properties (Gál et al., 2021).

Galectin-7 knockout mice recapitulated the phenotype of galectin-1-deficient mice in respect of the reduced wound closure efficiency. The experiments conducted with galectin-7-deficient cells suggested that the lack of this protein negatively affected the formation and/or stabilization of lamellipodia reducing thereby their motility potential (Gendronneau et al., 2008).

### 2.8 R-spondin family

The Roof Plate specific Spondin (RSPO (also named R-spondin)) protein family consists of four secreted proteins: R-spondin 1, R-spondin 2, R-spondin 3, and R-spondin 4. The structure of R-spondins is conserved: they consist of an N-terminal signal peptide, 2 cysteine-rich furin-like domains, a thrombospondin domain and a basic amino acid rich C-terminal domain (de Lau et al., 2012; Ter Steege and Bakker, 2021). The R-spondins enhance the signals mediated of the Wnt/β-catenin pathway, which regulates multiple fundamental processes including proliferation, stem cell control, tissue homeostasis and regeneration. R-spondin receptors are G-protein-coupled proteins that interact with their ligands through a leucine-rich repeat (LRP). These are present in multiple organs and regulate a spectrum of stem cell functions (see (de Lau et al., 2012; Ter Steege and Bakker, 2021) for comprehensive reviews).

RSPO1, also known as cysteine-rich single thrombospondin domain containing protein 3 or Cristin 3, is a 27 kDa secreted protein and possesses growth factor properties (Sundaramurthi et al., 2012). RSPO1 has been found to accelerate cutaneous wound healing in rats by stimulating angiogenesis and decreasing inflammation (Kuppan et al., 2011; Sundaramurthi et al., 2012). Recently, it was discovered that R-spondin-1 is secreted by fibroblasts and regulates the growth and/or differentiation of keratinocytes. The skin of a RSPO1-deficient patient who suffered from palmoplantar keratoderma displayed dysregulation of skin microenvironment and loss epidermal integrity that was suggested to increase the risk of squamous cell carcinoma (Dellambra et al., 2021; Dellambra et al., 2022).

Unlike RSPO1, RSPO2 is required for limb, lung and hair follicle development (de Lau et al., 2012). In a mouse model, exogenously administered RSPO2 activated hair follicle stem cells and promoted hair growth (Smith et al., 2016). Furthermore, RSPO2 was expressed at higher levels in cells derived from keloid tissue than in cells that originated from normal tissue suggesting a role for this protein in regulating the proliferation of fibroblasts (Chua et al., 2011). However, its role in wound healing has not yet been studied. Similarly, RSPO3 was found to be involved in the regulation of skin thickness, fibrosis, and collagen deposition (Zhang M. et al., 2020). To our best knowledge, the expression and role of RSPO4 in skin has not been addressed so far.

### 2.9 Fibulin family

The fibulin family of proteins share the C-terminal fibulin module and calcium-binding EGF-like domains. Eight members of this family have been identified in mammals: fibulin-1, -2, -3, -4, -5, -7, as well as hemicentin-1 and -2. Fibulin-3, -4, and -5 play crucial roles in elastic fiber assembly (Nakamura, 2018). These proteins interact with the components of the basement membrane and other ECM proteins, bind to elastin, latent TGF-β binding protein 4, and lysyl oxidase indirectly modulate the behavior of cells (Timpl et al., 2003; Nakamura, 2018). Fibulin-2 and fibulin-5 may directly bind integrins on cell surface and fibulin-1 interacts with the cytoplasmic fibronectin receptor β-subunit exerting thereby direct signaling cues to the cells (Argraves et al., 1989; Timpl et al., 2003) [see (Timpl et al., 2003) for comprehensive review].

Fibulin-1, is a component of the basal membrane and elastic fibers in many tissues including skin (de Vega et al., 2009). Fibulin-1 has been shown to regulate lung remodeling in mouse models of pulmonary disease (Liu et al., 2016) and upregulation of fibulin-1 has been observed in a mouse model of cardiomyopathy (Redfern et al., 2000) suggesting a role for this protein in tissue regeneration. Fibulin-2 is a secreted glycoprotein that is associated with embryonic development and tissue remodeling (Pan et al., 1993). Fibulin-2 knockout mice displayed perinatal skin blisters, which indicated a role for fibulin-2 in the maintenance of basal membrane integrity (Longmate et al., 2014). Fibulin-3 has low expression in normal skin keratinocytes but is upregulated in psoriasis (Wang X. et al., 2019). Fibulin-4 has been shown to be expressed in papillary dermis of human skin (Cristóbal et al., 2018). The role of fibulin-1 to 4 in the skin has not yet been characterized.

Fibulin-5 is normally expressed in reticular and papillary dermis; however, its expression decreases with aging and ultraviolet B exposure (Kadoya et al., 2005). In addition, fibulin-5 has a crucial role in elastic fiber formation *in vivo* as it binds elastin and links elastic fibers to other cell structures (Nakamura et al., 2002; Yanagisawa et al., 2002). Fibulin-5 plays important roles in every step of elastic fiber assembly (tropoelastin coacervation, recruitment of elastin microaggregates to microfibrils, and crosslinking of elastin), but has been shown that can be an agonist for β1 integrins in cell attachment, and antagonist in MMP and reactive oxygen species production (Nakamura, 2018). Overexpression of Fibulin-5 was shown to promote wound healing *in vivo* in rabbits with full-thickness dermal wounds (Lee et al., 2004). Although an upregulation of fibulin-5 expression in the granulation tissue of full-thickness cutaneous wounds was observed in wild-type mouse skin, no difference in the wound closure rate between fibulin-5-deficient and wild-type mouse skin was detected. Concomitantly, it was established that fibulin-5 did not regulate the proliferation and migration of fibroblasts (Zheng et al., 2006).

### 2.10 Ecto-nucleotide pyrophosphatase/phosphodiesterase family

The first member of ENPP family, the ENPP1 protein, was discovered more than 50 years ago, and the for a long time generation, breakdown and recycling of extracellular nucleotides were considered as the main functions of ENPP proteins (Massé et al., 2010). Currently, the ENPP protein family encompasses seven members (ENPP1-7) and is characterized by the presence of a conserved phosphodiesterase domain. The most well-characterized proteins of this family are ENPP1 and ENPP2 ENPPs are transmembrane ecto-enzymes but only ENPP2 or autotaxin is a secreted protein and can thus be considered a matricellular protein (Cholia et al., 2015; Onyedibe et al., 2019; Borza et al., 2022). ENPP2 possesses lysophospholipase properties and hydrolyses extracellular lysophosphatidylcholines producing lysophosphatidic acids (LPAs) that activate various signaling pathways, exerting its function indirectly in the cells (Borza et al., 2022). ENPP2 participates in regulation of the homeostasis of several tissue and participates in a variety of pathological processes including tumor progression, immune evasion, T-cell migration, inflammation, and wound healing (Borza et al., 2022).

ENPP2 is highly expressed in the postnatal dermal papilla, but it is dispensable for hair follicle formation, and its ablation in knockout mice is embryonically lethal due to impaired development of blood vessels (Grisanti et al., 2013). Interestingly, LPA administration in different wound models stimulated the proliferation and differentiation of cells as well as facilitated wound re-epithelialization suggesting a possible stimulatory role for this protein in cutaneous wound healing (Piazza et al., 1995; Demoyer et al., 2000; Balazs et al., 2001). As ENPP2 regulates the migration of mesenchymal stem cells derived from human umbilical cord blood, stimulation of fibroblast proliferation and migration may be the cellular mechanisms by which ENPP2 exerts its effects. At the molecular level it has been shown that ENPP2-LPA signaling disrupts adherent junctions and causes rearrangement of cytoskeleton through LPA receptor 1/3-dependent PKC/GSK-3β/β-catenin and PKC/Rho GTPase pathways (Ryu and Han, 2015). In addition, the expression of ENPP2 was higher in blistering skin than in normal skin suggesting a role for this protein in maintaining epidermal integrity (Mazereeuw-Hautier et al., 2005). Nevertheless, a defined role for ENPP2-LPA axis in cutaneous wound healing is yet to be established.

### 2.11 Olfactomedin family

Olfactomedin was discovered in the early 1990s among a large number of glycosylated proteins that were found to be present in the olfactory neuroepithelium of a bullfrog, hence the name (Snyder et al., 1991). Since then, 13 proteins have been identified that share the 250 amino acid olfactomedin domain and form the olfactomedin protein family (Zeng et al., 2005). In both humans and mice, the olfactomedin family consists of olfactomedin (OLFM) 1-4, adhesion G protein-coupled receptor L (ADGRL) ADGRL1, ADGRL2 and ADGRL3, myocilin, gliomedin as well as of olfactomedin-like proteins (OLFML) 1, 2A, 2B, and 3 (Zeng et al., 2005). OLFM1 is expressed mainly in the brain, OLFM2 in the pancreas and prostate, OLFM3 in the cerebellum, OLFM4 in the colon, small intestine as well as in prostate, and Myocilin in the heart and skeletal muscle (Kulkarni et al., 2000). Initially, mutations in the Myocilin (previously named trabecular meshwork-induced glucocorticoid protein or TIGR), and olfm2 (previously named Noelin2) genes were associated with primary open glaucoma (Stone et al., 1997; Mukhopadhyay et al., 2004). The studies conducted in this area lead eventually to the characterization of the whole olfactomedin protein family (Wang H. et al., 2019; Tanji et al., 2021).

Only a few members of the OLFM family have been associated with skin maintenance and cutaneous wound healing. OLFM4 is mainly expressed in prostate, small intestine, colon, bone marrow, and stomach as well as in several forms of cancer (Tomarev and Nakaya, 2009; Guette et al., 2015; Chen Z. et al., 2022). Nevertheless, OLFM4 also plays an important role in innate immunity against bacterial infection, regulates gastrointestinal inflammation (Liu and Rodgers, 2016) and is a promising biomarker for certain viral and bacterial infections (Liu and Rodgers, 2022). Our group found that OLFM4 stimulated keratinocyte proliferation and enhanced keratinocyte and fibroblast migration *via* downregulating PTEN and activating POU5F1/OCT4 signaling *in vitro*. Furthermore, topical administration of OLFM4 protein accelerated cutaneous wound healing in mice. Interestingly, the increase in OLFM4 level was detected in hyperproliferative lesions of the psoriasis patients suggesting that stimulation of keratinocyte proliferation may underlie the stimulatory effects of OLFM4 on wound healing (Klaas et al., 2022). Furthermore, studies on incisional wound healing in rabbits suggest that OLFM4 may also have a role in healing the mucosal injury (Gallant-Behm et al., 2011; Dolivo et al., 2022).

Initially, OLFML3 was considered a regulator of embryonic patterning *via* recruitment of bone morphogenetic protein 1 and interaction with Notch and Wnt pathways (Inomata et al., 2008). Later it was found that OLFML3-knockout mouse showed defects in vascular remodeling under normal and pathological conditions and it was proposed that modulation of bone morphogenetic protein 4 and SMAD1/5/8 signaling by OLFML3 had an impact on the activation of endothelial cells (Miljkovic-Licina et al., 2012; Imhof et al., 2020). An elegant series of experiments convincingly demonstrated the proangiogenic effect of OLFML3 on mouse and rat wound healing. The application of recombinant OLFML3 to full-thickness cutaneous wounds markedly accelerated the wound closure and similar positive effects were observed when subcutaneous implantation of OLFML3 coated electrospun poly (ɛ-caprolactone) scaffolds were used to modulate cutaneous wound healing. Increased migration and attachment of endothelial cells was shown to be the major process that contributed to improved wound healing (Dunn et al., 2016).

OLFML2A was overexpressed in the skin of premenopausal Chinese Han women compared with postmenopausal women (Yan et al., 2011). Downregulation of OLFML2A in MDA-MB-231 triple negative breast cancer cells decreased their migration, proliferation, and invasion (Gao et al., 2022). However, the role of OLFML2A in regulation of cutaneous wound healing has yet to be established.

## 3 Conclusion

Research over 2 decades has focused on capturing the roles of matricellular proteins in a variety of physiological and pathological processes. While they are important regulators of organ development in the embryo and fetus, during adulthood, they are mostly expressed at low levels in tissues. However, after and during periods of stress such as acute and chronic inflammation and various types of injury, their expression is again increased. Accordingly, research has allocated key roles for matricellular proteins in processes striving for tissue repair, regeneration, and regaining tissue homeostasis. Some of these actions, such as formation of scars and fibrosis, can even be considered acutely critical for survival of the individual after organ damage. Matricellular proteins are also important regulators of angiogenesis, inflammation, and ECM formation as well as, in general, guiding the rebuilding of tissue. Their expression is regulated in tissue- and cell-type-dependent manners, and they exert their actions through interactions with cellular receptors. Importantly, the effects of matricellular proteins are modified by their environment and binding partners. Revealing these interactions is key to deciphering the complexity of matricellular protein signaling. Not only is this increasing mechanistic knowledge helpful for interpretation of existing and emerging results as well as guiding the design of experimentation, but it also aids in and improves the development of therapeutic approaches based on the matricellular signaling concept.

Although the complexity of wound healing is appreciated, our current understanding falls short in the appreciation of the level of intricate detail at which different cellular and acellular skin components drive the progression of wound healing. It will be important to combine a network of molecular and cellular interactions and regulome of wound healing with the involvement of the matricellular and the ECM and its matricryptin signaling networks for development of intelligent wound dressings, biologicals, and small-molecule drugs for driving effective functional skin wound healing and limiting scarring.

## Abbreviations

CCN, cellular communication network; TGF, transforming growth factor; THBS, thrombospondins; MMP, matrix metalloprotease; EGF, epidermal growth factor; TN-, tenascin; TGFBI, transforming growth factor beta induced protein; SMOC, secreted modular calcium binding protein; FSTL1, follistatin like protein 1; SIBLING, Small Integrin-Binding Ligand, N-linked Glycoproteins; OPN, osteopontin; CRD, carbohydrate-recognition domain; RSPO, Roof Plate specific Spondin protein; ENPP, ectonucleotide pyrophosphatase/phosphodiesterases; LPA, lysophosphatidic acids; ADGRL, adhesion G protein-coupled receptor L; OLFM, olfactomedin protein; OLFML, olfactomedin-like proteins.
